# Maternal psychological distress and temperament traits in children from infancy to late childhood

**DOI:** 10.1002/jcv2.12242

**Published:** 2024-05-06

**Authors:** Marius Lahti‐Pulkkinen, Anna Lähdepuro, Jari Lahti, Polina Girchenko, Riikka Pyhälä, Rebecca M. Reynolds, Pia M. Villa, Hannele Laivuori, Eero Kajantie, Kati Heinonen, Katri Räikkönen

**Affiliations:** ^1^ Faculty of Medicine Department of Psychology and Logopedics University of Helsinki and Helsinki University Central Hospital Helsinki Finland; ^2^ The Population Health Unit The Finnish Institute for Health and Welfare Helsinki Finland; ^3^ Centre for Cardiovascular Science University of Edinburgh Edinburgh UK; ^4^ Yale Child Study Center Yale University New Haven Connecticut USA; ^5^ Obstetrics and Gynecology University of Helsinki and Helsinki University Hospital Helsinki Finland; ^6^ Medical and Clinical Genetics University of Helsinki and Helsinki University Hospital Helsinki Finland; ^7^ Institute for Molecular Medicine Finland Helsinki Institute of Life Science University of Helsinki Helsinki Finland; ^8^ Faculty of Medicine and Health Technology Department of Obstetrics and Gynecology Tampere University Hospital and Center for Child Adolescent and Maternal Health Research Tampere University Tampere Finland; ^9^ Clinical Medicine Research Unit MRC Oulu Oulu University Hospital and University of Oulu Oulu Finland; ^10^ Children's Hospital Helsinki University Hospital and University of Helsinki Helsinki Finland; ^11^ Department of Clinical and Molecular Medicine Norwegian University for Science and Technology Trondheim Norway; ^12^ Welfare Sciences Faculty of Social Sciences University of Tampere Tampere Finland; ^13^ Department of Obstetrics and Gynecology Helsinki University Hospital and University of Helsinki Helsinki Finland

**Keywords:** anxiety, depression, prenatal, stress, temperament

## Abstract

**Background:**

Maternal psychological distress during pregnancy is associated with infant temperament. Whether associations persist into late childhood, whether maternal distress is associated with temperament change from infancy to late childhood, whether associations are independent of maternal concurrent distress, and whether maternal distress has sensitive exposure periods on child temperament remain unclear.

**Methods:**

Our study includes mother‐child dyads from Finnish, prospective Prediction and Prevention of Preeclampsia and Intrauterine Growth Restriction study. The mothers completed the Center for Epidemiologic Studies Depression Scale, State Anxiety Inventory and Perceived Stress Scale: biweekly up to 14 times during pregnancy; once in infancy (at child age 4–12 months); and once in late childhood (at child age 7–11 years). They also completed the Infant Behavior Questionnaire Revised at the infancy (*n* = 2538) and Temperament in Middle Childhood Questionnaire at the late childhood (*n* = 2004; 1693 children had data at both follow‐ups) follow‐up on child negative affectivity, extraversion and effortful control. We examined the associations of maternal distress with child temperament with linear regression, linear mixed and Bayesian relevant lifecourse exposure models.

**Results:**

Maternal distress during pregnancy was associated with higher negative affectivity and lower effortful control in children in infancy and late childhood. Maternal distress during pregnancy was also associated with increases in negative affectivity, decreases in effortful Control, and smaller decreases in extraversion from infancy to late childhood. The associations with late childhood temperament and temperament change were independent of maternal concurrent distress. Late childhood was a sensitive period for lifetime‐to‐date effects of maternal distress on late childhood negative affectivity and effortful control. Distress during pregnancy and infancy had smaller contributions.

**Conclusions:**

Maternal psychological distress during pregnancy is associated with individual differences and change in child temperament from infancy to late childhood. However, distress during pregnancy has a smaller effect on late childhood temperament than maternal concurrent distress.

## Graphical abstract

We examined among over 2000 mother‐child dyads the associations of maternal psychological distress (depressive, anxiety and perceived stress symptoms) during pregnancy with child temperament traits in infancy and in late childhood. In our longitudinal study, maternal psychological distress during pregnancy was associated with higher negative affectivity and lower Effortful Control in children in infancy and in late childhood and with changes in these temperament traits from infancy to late childhood.

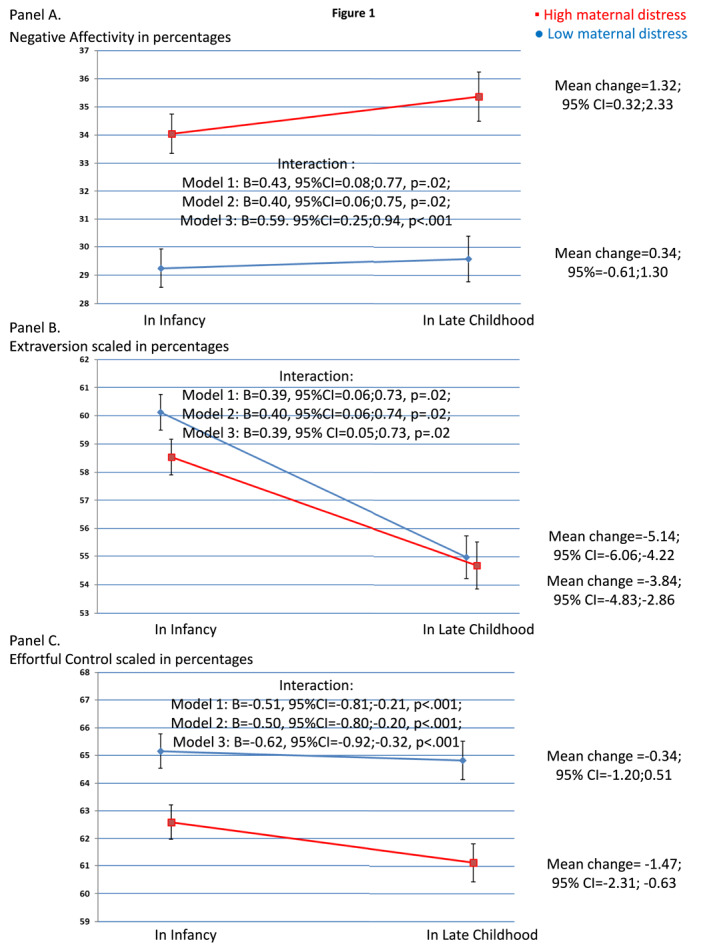

**Key points**

**What's known?**

- Meta‐analyses have shown that maternal psychological distress during pregnancy is associated with infant temperament.

**What's new?**

- Our novel, prospective study shows that these associations persist to late childhood, and that maternal psychological distress during pregnancy is also associated with change in child temperament traits from infancy to late childhood.
- Maternal psychological distress has lifetime‐to‐date effects on late childhood temperament, where distress in the late childhood period contributes the most, but distress during pregnancy and in infancy also have effects.

**What's relevant?**

- The associations of maternal psychological distress with child temperament traits, in particular with higher negative affectivity and lower effortful control, persist from infancy to late childhood.

## INTRODUCTION

The psychobiological model of temperament defines temperament as constitutional, biology‐based individual differences in reactivity and self‐regulation, which show both stability and change during development and are expressed along three continuous dimensions: negative affectivity, extraversion and effortful control (Komsi et al., 2006; Putnam et al., 2008; Rothbart & Derryberry, 1981; Rothbart et al., 2001). Individual differences in these early emerging temperament traits, specifically in negative affectivity and effortful control are key determinants of internalizing and externalizing psychopathology (Kostyrka‐Allchorne et al., 2020).

Both genetics and exposure to environmental adversities during sensitive periods of brain development may underlie individual differences in temperament (Guo et al., 2011; Mikolajewski et al., 2013; Takegata et al., 2021; Van den Bergh et al., 2020; Yamagata et al., 2005). An adversity combining shared genetic risks for negative affectivity and internalizing psychopathology with prenatal environmental adversities is maternal psychological distress during pregnancy (Ahmadzadeh et al., 2021; Chen et al., 2023; Gjerde et al., 2017; Hannigan et al., 2018; Mikolajewski et al., 2013; Perlstein & Waller, 2022). Psychological distress is defined here as depressive, anxiety and perceived stress symptoms (Hicks et al., 2019; Tuovinen et al., 2021). There is meta‐analytic evidence that maternal psychological distress during pregnancy is associated with significantly higher negative affectivity and marginally lower effortful control in infancy (Rogers et al., 2020; Spry, Aarsman, et al., 2020). However data remain scarce on the associations with temperament traits assessed after infancy.

We know of only five studies, which examined the associations of maternal psychological distress during pregnancy and temperament traits in children in early or late childhood. Four of these studies used a prospective design (Glynn et al., 2018; Mahrer et al., 2020; Rinne et al., 2022; Swales et al., 2023). Three such studies reported on the associations of different indicators of maternal psychological distress during pregnancy with temperament traits in 3–5 year‐old children among 95–125 mother‐child dyads of the same prospective study cohort (Mahrer et al., 2020; Rinne et al., 2022; Swales et al., 2023). In that cohort, maternal depressive symptoms during pregnancy were associated with higher negative affectivity but not effortful control (Rinne et al., 2022; Swales et al., 2023). Maternal pregnancy‐specific anxiety during the second but not third pregnancy trimester was also associated with higher negative affectivity (Mahrer et al., 2020). In contrast, maternal perceived stress symptoms were not associated with negative affectivity or effortful control (Swales et al., 2023). This cohort benefited from an ethnically and socioeconomically diverse study sample (Mahrer et al., 2020; Rinne et al., 2022; Swales et al., 2023). In another prospective study among 257 mother‐child dyads, a combined scale of maternal depressive, anxiety, perceived stress and pregnancy‐specific stress symptoms was associated with increased negative affectivity in 7‐year‐old children (Glynn et al., 2018). The generalizability of that study was strengthened by combining data from two prospective study cohorts (Glynn et al., 2018). In another study of 3735 mother‐child dyads, maternal psychological distress during pregnancy was associated with higher negative‐affectivity‐related reactivity and lower effortful‐control‐related persistence, but not with extraversion‐related sociability in 4–7 and 8‐15‐year‐old children (Sutin et al., 2022). The strengths of that study included the largest sample size to date on maternal distress and late childhood temperament and the repeated temperament assessments with corresponding results (Sutin et al., 2022). However, in that study maternal psychological distress during pregnancy was retrospectively reported after childbirth with one item indicating problems with depression, anxiety or stress (Sutin et al., 2022).

Importantly, we know of no studies examining whether maternal psychological distress during pregnancy is associated with change in child temperament from infancy to later childhood, although temperament traits also change due to maturation and experience (Briley & Tucker‐Drob, 2014). Furthermore, although meta‐analysis shows that maternal distress after pregnancy is also associated with higher negative affectivity and lower effortful control in children (Rogers et al., 2020), evidence remains inconclusive on whether maternal psychological distress during pregnancy is associated with child temperament independently of maternal concurrent distress (Korja et al., 2017; Sutin et al., 2022). In addition, the presence or absence of sensitive exposure‐to‐adversity periods for psychological development has been emphasized as an important study question in recent scientific literature (Davis & Narayan, 2020; Gabard‐Durnam & Mclaughlin, 2019; Madathil et al., 2018; Woodard & Pollak, 2020). However, we know of no studies directly comparing the relative importance of different exposure periods by examining whether there are sensitive or critical exposure periods for the associations of maternal psychological distress during pregnancy, child's infancy or late childhood with child temperament, or whether the effects of maternal distress on child temperament solely accumulate across time (Gabard‐Durnam & Mclaughlin, 2019; Madathil et al., 2018). The existence of a sensitive period would mean that exposure during one period is more important than at another also relevant period. A critical period indicates that exposure only during a certain period matters, whereas accumulative effects suggest equal effects for all periods (Gabard‐Durnam & Mclaughlin, 2019; Madathil et al., 2018).

To address these knowledge gaps, we examined whether maternal psychological distress during pregnancy was prospectively associated with negative affectivity, extraversion and effortful control in children both in the infancy (age 4–12 months) and late childhood (age 7–11 years) periods, and with change in these temperament traits between infancy and late childhood. We also examined whether any associations were independent of maternal concurrent psychological distress when rating the child temperament. By using Bayesian relevant lifecourse exposure models (BRLM), we also studied whether there are sensitive or critical periods either during pregnancy, in child's infancy or late childhood or accumulative effects across time for possible lifetime‐to‐date effects of maternal psychological distress on child temperament in late childhood (Madathil et al., 2018). The BRLM compares the relative contributions of an exposure at different developmental periods on an outcome, and examines whether critical period, sensitive period or accumulative effect models best describe possible lifetime‐to‐date effects of an exposure on an outcome (Madathil et al., 2018). The BRLM is designed to compare the effects of highly correlated exposures, for example, maternal psychological distress, across time, avoiding multicollinearity bias which may be present in mutually adjusted linear regression models (Madathil et al., 2018). Furthermore, previous evidence suggests possibly sex‐specific associations of maternal psychological distress with child temperament (Savory et al., 2020). Hence, we tested whether associations between maternal distress and child temperament differed by child sex.

## METHODS

Our study cohort, the Prediction and Prevention of Preeclampsia and Intrauterine Growth Restriction (PREDO), is a prospective pregnancy cohort of 4777 women and their singleton children born alive between 2006 and 2010. The cohort profile provides the details of the study design (Girchenko et al., 2017). Women were recruited to the study in early pregnancy at their first ultrasound screens at 10 different maternity hospitals in Southern and Eastern Finland. We obtained written informed consent forms from all participating women. Three women have since withdrawn consent. Of the 4774 remaining women, 3399 (71.2%) completed biweekly questionnaires on their depressive, anxiety, and perceived stress symptoms during pregnancy from gestational weeks + days 12 + 0/13 + 6 until 38 + 0/39 + 6/childbirth.

After childbirth, the mothers and their children were invited for follow‐up studies at child age of 4–12 months (median age = 6.3 months, interquartile range [IQR] = 6.0–6.6 months, 10–90 percentile range = 5.8–7.3 months) and of 7–11 years (median age = 9.4 years, IQR = 8.9–10.1 years, 10–90 percentile range = 8.3–10.3 years). In these two follow‐ups, the mothers completed the same scales of their depressive, anxiety and perceived stress symptoms as during pregnancy, and additionally, questionnaires on child temperament.

Of the 3399 women with data on maternal psychological distress during pregnancy, 2538 (74.7%) provided data on child temperament in infancy, and 2004 (59.0%) in late childhood. These mother‐child dyads comprised the samples to study whether maternal psychological distress during pregnancy was associated with child temperament traits in infancy and late childhood, respectively. For the analyses of change in child temperament from infancy to late childhood, the analytic sample comprised 1693 (49.8 of the initial sample with psychological distress data) mother‐child days with data on maternal psychological distress during pregnancy and on child temperament in both the infancy and late childhood follow‐ups. To study the sensitive exposure periods of the effects of maternal psychological distress on late childhood temperament, the analytic sample comprised 1926 (56.7%) mothers for whom we had data on psychological distress during pregnancy, infancy and late childhood, and of whose children we had temperament data during late childhood.

Table S1 shows the differences between the participating and non‐participating subsamples of the cohort. Compared to the non‐participating mothers, the mothers belonging to the different analytic subsamples reported lower perceived stress and/or anxiety symptoms during pregnancy. They also had higher education levels, were older at childbirth and more often primiparous, and had less often had cardiometabolic pregnancy disorders or lifetime‐to‐date mental and behavioral disorders. The children of the mothers participating in both follow‐ups include a larger proportion of girls than the children of the non‐participating mothers (Table S1).

### Ethical considerations

The PREDO study protocol has been approved by the Ethics Committee of the Helsinki and Uusimaa Hospital District and by the participating study hospitals (Girchenko et al., 2017). As stated, all women participating in the study signed written informed consent forms. Since our study data includes sensitive patient data, we cannot make it open access. Researchers interested in data access may contact the PREDO study board. Data requests will be subject to further reviews by relevant register authorities.

### Maternal psychological distress during and after pregnancy

Between pregnancy weeks 12/13 and 38/39/delivery, the women rated their depressive symptoms with the Center for Epidemiologic Studies Depression Scale (CES‐D), anxiety symptoms with the State version of the State‐Trait Anxiety Inventory (STAI), and perceived stress symptoms with a 5‐item version of the Perceived Stress Scale (PSS) (Cohen et al., 1983; Radloff, 1977; Spielberger et al., 1983). The women completed these questionnaires biweekly, up to 14 times during pregnancy.

The mothers completed these same questionnaires during the follow‐ups in the child's infancy and in late childhood. For 28 mothers with missing data on distress at the infancy follow‐up (but with data on child temperament in infancy), we imputed data on maternal distress from an earlier follow‐up at 0.6–18.0 weeks of child age (median = 2.2, SD = 3.1, IQR = 2.0–2.6, 10–90 percentile range = 1.8–4.2 weeks).

The CES‐D, STAI and PSS are validated in the general population (Lee, 2012; Spielberger et al., 1983; Vilagut et al., 2016). All three scales have been validated also among pregnant women (Heller et al., 2022; Karam et al., 2012; Tendais et al., 2014; Tuovinen et al., 2021). In our sample, the internal consistencies (Cronbach alphas) during pregnancy and at the infancy and late childhood follow‐ups ranged from 0.89 to 0.92 for CES‐D, from 0.74 to 0.78 for PSS, and from 0.94 to 0.95 for STAI. Since depressive (Pearson correlation coefficients [*r*] = .50 to .80, *p* < 0.001), anxiety (*r* = .42 to .69, *p* < .001) and perceived stress (*r* = .44 to .71, *p* < .001) symptoms were highly stable across pregnancy, we used trimester‐weighted mean scores of these symptom scales across pregnancy in our analyses (Lahti et al., 2017; Tuovinen et al., 2021).

### Temperament traits in children

The mothers rated their children's temperament traits in the infancy follow‐up with the Infant Behavior Questionnaire Revised (IBQ‐R) and in the late childhood follow‐up with the Temperament in Middle Childhood Questionnaire (TMCQ) (Gartstein & Rothbart, 2003; Simonds, 2006). Although the temperament factor scales have age‐specific names in IBQ‐R and TMCQ, they address the same overarching temperament dimensions in an age‐specific manner (Rothbart, 2007; Shiner et al., 2012). Hence, we refer to these factors hereafter at both follow‐ups as negative affectivity, extraversion and effortful control.

The IBQ‐R comprises 191 items on the frequency of infant's reactions in specific types of situations which are rated from 1 (never) to 7 (all of the time) (Gartstein & Rothbart, 2003). This well‐validated questionnaire has 14 subscales, which load onto three factors; negative affectivity, extraversion and effortful control (Dias et al., 2021; Gartstein & Rothbart, 2003; Parade & Leerkes, 2008; Toffol et al., 2019). The negative affectivity factor includes 59 items, the extraversion factor comprises 72 items, and the effortful control factor consists of 60 items. In our sample, the internal consistencies of all three factors were high: Cronbach's *α* = .93 for negative affectivity, *α* = .91 for extraversion, and *α* = .90 for effortful control.

The TMCQ includes 157 statements regarding child's behavior, which are rated from 1 (basically never true) to 5 (almost always true) (Simonds, 2006). A factor‐analytic study in a Nordic country has shown that the TMCQ yields 14 subscales which load onto three factors: negative affectivity (including 44 items), extraversion (including 38 items) and effortful control (including 48 items) (Nystrom & Bengtsson, 2017). In our sample, these factors showed high internal consistencies: Cronbach's *α* = .92 for negative affectivity, *α* = .90 for extraversion, and *α* = .89 for effortful control.

To enable assessment of changes in these age‐specific temperament trait scores from infancy to late childhood, we present the temperament trait scores in infancy and late childhood in percentage units. First, a constant of one, the minimum score for the scale, was subtracted from the score of each child, yielding scores ranging from zero to six for IBQ‐R and zero to four for TMCQ. Then, these scores were divided by six (maximum score‐1) for IBQ‐R and by four (maximum score‐1) for TMCQ, and thereafter multiplied by 100, to yield percentage unit scores with possible values between zero and 100. A higher percentage unit score indicates that the score is closer to the maximum.

### Covariates

We selected covariates based on their previously reported associations with maternal psychological distress and/or child temperament (Cassiano et al., 2020; Froggatt et al., 2020; Spry, Moreno‐Betancur, et al., 2020; Takegata et al., 2021; Thiel et al., 2020). Of the covariates, data on maternal age at childbirth, parity and child sex, birth weight and gestation length (preterm birth vs. other) came from Finnish Medical Birth Register (MBR). Maternal substance use (yes/no) during early pregnancy was identified combining data on smoking from the MBR with self‐report data on alcohol use reported in early pregnancy. The mothers self‐reported their highest achieved education level (primary or secondary vs. tertiary) concurrent to the child temperament trait ratings in late childhood. Child age at follow‐up was calculated by subtracting the date of the completion of the temperament questionnaire (reported by the mother) from the birth date of the child (extracted from the MBR).

Furthermore, we identified maternal lifetime‐to‐date mental and behavioral disorders from Care Register for Health Care (HILMO), a validated register covering diagnoses of all hospitalizations in Finland and all outpatient visits in public specialized medical care in Finland (Sund, 2012). We identified mental and behavioral disorder diagnoses with International Classification of Diseases (ICD), 8th, 9th and 10th Revision codes 290–315, 290–319 and F00‐F99, respectively. We identified maternal cardiometabolic pregnancy disorders combining data from the MBR, HILMO and medical records. Mothers were grouped into three categories: (a) mothers who had cardiometabolic conditions in current pregnancy (mothers who were overweight or obese [body mass index [weight (kg)/height (m)^2^]] ≥ 25) in early pregnancy or had gestational, type I or type 2 diabetes (ICD‐9 codes 2500–2509 and ICD‐10 codes E10–E11 and O24) or preeclampsia, chronic, gestational or unspecified hypertension (ICD‐10: O10–O11, O13–O16, and I10), (b) mothers who had hypertension or diabetes only in previous pregnancy (ICD‐9: 401–405, 642, 6480A, 6488A; ICD‐10: O10–O11, O13–O16, O24; I10), (c) mothers who had none of these conditions (Lahti‐Pulkkinen et al., 2020).

### Statistical analyses

Maternal depressive, anxiety and stress symptoms showed strong concurrent associations with each other during pregnancy, infancy and late childhood (Pearson *r*:s ranging from .64 to .84, all *p*‐values <.001). Hence, at each time point (during pregnancy, infancy and late childhood) we ran principal component analyses (PCA) to reduce the number of dimensions of these symptom scales and reduce the likelihood of type 1 error due to multiple testing in our analyses. Table S2 shows that during pregnancy, infancy, and late childhood, the PCA yielded one‐component solutions explaining 88%, 81%, and 78% of the variance, respectively (Table S2). For each follow‐up, we thus created one PCA score, reflecting maternal psychological distress. This psychological distress PCA score showed high consistency from pregnancy to child's infancy and late childhood (Pearson *r* = .66 for the association between PCA scores during pregnancy and child's infancy, *r* = .51 between pregnancy and late childhood, and *r* = .49 between infancy and late childhood; all *p*‐values ≤.001).

All maternal psychological distress scale scores during pregnancy, child's infancy and late childhood were square root transformed and standardized before conducting the PCA on them. After conducting the PCA, the obtained PCA scores were standardized to facilitate the interpretation of effect sizes. Child temperament trait scores followed normal distributions.

To address our main study questions, we first examined with linear regressions the associations of the maternal psychological distress PCA scores during pregnancy and concurrently to child temperament ratings with negative affectivity, extraversion, and effortful control in children in infancy and late childhood. Next, we examined with linear mixed models whether maternal psychological distress PCA score during pregnancy was associated with change in child temperament traits from infancy to late childhood. The mixed models included negative affectivity, extraversion, and effortful control as the within‐person outcome variables (each tested in a separate model), child's age at testing as a within‐person predictor and maternal distress PCA score during pregnancy as the between‐person fixed effect. Through the interaction of child's age at assessment *x* maternal distress PCA score we tested whether maternal distress PCA score during pregnancy was associated with change in child temperament traits from infancy to late childhood. Significant interactions were depicted in groups divided by the median value of the maternal distress PCA score during pregnancy.

In the linear regression and mixed models, we first made adjustments for child sex and age (model 1), then included also maternal age, education level, parity, substance use in early pregnancy, cardiometabolic pregnancy disorders, and lifetime‐to‐date mental and behavioral disorders by child follow‐up, and child preterm versus term birth and birth weight in the second model (model 2), and finally included also the maternal concurrent psychological distress PCA score (model 3). When examining the main effects of maternal concurrent distress, the third regression models were adjusted for maternal distress during pregnancy.

Next, we examined with BRLM the lifetime‐to‐date effects and potential sensitive or critical periods or accumulative effects of maternal psychological distress, assessed during pregnancy, infancy, and late childhood, on child temperament traits in late childhood (Madathil et al., 2018). Before running these analyses, we tested with linear regression analyses whether maternal distress PCA score during pregnancy, infancy and late childhood was associated with child negative affectivity, extraversion, and effortful control in late childhood among the sensitive period analytic subsample, and whether the associations were in the same direction for all exposure periods (an underlying assumption of the BRLM [Madathil et al., 2018]). Then, using BRLM, we assessed with deltas and 95% Credible Intervals (CrI) whether maternal distress PCA scores had lifetime‐to‐date effects on child temperament traits in late childhood. If lifetime‐to‐date effects were present, we tested also with BRLM with weights and 95% CrI the relative contributions of each exposure period and examined with Euclidean Distances and posterior probabilities which of the models (the sensitive period, critical period or accumulative effect model) provided the best fit with the observed data (Madathil et al., 2018). We present the weights in percentage units to facilitate their interpretation. The sensitive period model was hypothesized to have a weight of 66.7% for one exposure period and weights of 16.7% for the two other periods. The accumulative effect model had expected weights of 33.3% for all three periods, while the critical period model had a hypothesized weight of 100.0% for one period (Madathil et al., 2018). The BRLM analyses were adjusted for child sex and age.

Finally, we examined whether any associations varied by child sex by adding interaction terms of maternal psychological distress PCA score during pregnancy and child sex to the abovementioned linear regression, linear mixed model and BRLM models. The linear regression and mixed model analyses were performed with IBM SPSS Statistics 29.0. The BRLM analyses were conducted using the Rstan programming language in R 4.1.0.

## RESULTS

Table 1 shows the characteristics of our analytic samples. On average, negative affectivity scores increased (mean difference [MD] = 0.91% units, 95% confidence interval [CI] = 0.13; 1.53) and extraversion (MD = −4.49% units, 95% CI = −5.16; −2.81) and effortful control (MD = −0.91% units, 95% CI = −1.50; −0.31) scores decreased from infancy to late childhood (Table 1; *p*‐values ≤.02). Table S3 shows that the temperament traits displayed significant but modest homotypic and heterotypic stability from infancy to late childhood. Table S4 shows that all assessed covariates except for maternal substance use during pregnancy showed significant associations with child temperament traits in infancy and/or late childhood.

**TABLE 1:** Sample characteristics. Means and standard deviations for continuous variables and number and percentage of participants for categorical variables in the subsamples with temperament data in child's infancy, late childhood or both (mixed models analytic sample), or with late childhood temperament data and maternal psychological distress data during pregnancy, and in child's infancy and late childhood (BRLM analytic sample).

	Temperament data in infancy	Temperament data in late childhood	Mixed models analytic sample	BRLM analysis analytic sample
*N*	2538	2004	1693	1926
Maternal characteristics	Mean (SD)/*N* (%)	Mean (SD)/*N* (%)	Mean (SD)/*N* (%)	
Psychological distress				
During pregnancy				
CES‐D depressive symptoms	11.4 (6.4)	11.2 (6.3)	11.2 (6.2)	11.2 (6.2)
STAI anxiety symptoms	33.0 (7.7)	32.7 (7.4)	32.7 (7.5)	32.7 (7.4)
PSS perceived stress symptoms	5.2 (2.5)	5.1 (2.4)	5.1 (2.4)	5.1 (2.4)
In child's infancy	(*n* = 2535)		(*n* = 1692)	
CES‐D depressive symptoms	9.4 (7.2)	‐	9.2 (7.1)	10.0 (6.7)
STAI anxiety symptoms	30.5 (8.5)	‐	30.4 (8.5)	31.8 (8.1)
PSS perceived stress symptoms	5.0 (3.1)	‐	4.9 (3.0)	5.1 (2.9)
Data missing	3	‐	1	0
In late childhood	‐	(*n* = 1976)		
CES‐D depressive symptoms	‐	8.9 (8.8)	8.9 (8.9)	8.8 (8.8)
STAI anxiety symptoms	‐	32.1 (9.5)	32.1 (9.8)	32.1 (9.6)
PSS perceived stress symptoms	‐	6.3 (3.6)	6.2 (3.7)	6.2 (3.6)
Data missing	‐	28	23	0
Age at delivery (years)	31.9 (4.7)	32.0 (4.5)	32.0 (4.5)	32.0 (4.5)
Parity, primiparous	1070 (42.2%)	817 (40.8%)	715 (42.2%)	791 (41.1%)
Cardiometabolic pregnancy disorders				
Overweight/obesity, diabetes or hypertensive disorder in current pregnancy	1078 (42.5%)	814 (40.6%)	687 (40.6%)	783 (40.7%)
Diabetes or hypertension only in previous pregnancy	94 (3.7%)	80 (4.0%)	61 (3.6%)	74 (3.8%)
No cardiometabolic disorder	1366 (53.8%)	1110 (55.4%)	945 (55.8%)	1069 (55.5%)
Alcohol use or smoking in early pregnancy, yes	508 (20.0%)	402 (20.1%)	327 (19.3%)	385 (20.0%)
Lifetime mental disorders, yes	216 (8.5%)	276 (13.8%)	243 (14.4%)	265 (13.8%)
Data missing	2	0	0	0
Education level				
Primary or secondary	774 (30.5%)	506 (25.2%)	421 (24.9%)	489 (25.4%)
Tertiary	1764 (69.5%)	1498 (74.8%)	1272 (75.1%)	1437 (74.6%)
Child characteristics				
Sex, girl	1297 (51.1%)	987 (49.3%)	847 (50.0%)	970 (50.4%)
Gestational age (weeks)	39.9 (1.5)	39.9 (1.5)	39.9 (1.5)	40.0 (1.5)
Preterm birth, yes	92 (3.6%)	75 (3.7%)	63 (3.7%)	71 (3.7%)
Birth weight (grams)	3524.6 (510.8)	3526.3 (511.3)	3527.1 (511.7)	3527.7 (511.8)
Data missing	9	5	3	3
Temperament trait scores in percentage units, a higher score indicates a score that is closer to the maximum score				
Negative affectivity				
In infancy	31.8 (10.6)	‐	31.6 (10.5)	‐
In late childhood	‐	32.5 (12.6)	32.5 (12.8)	32.4 (12.6)
Extraversion				
In infancy	59.9 (9.4)	‐	59.3 (9.4)	‐
In late childhood	‐	54.9 (11.9)	54.8 (11.8)	54.9 (11.8)
Effortful control				
In infancy	64.2 (9.4)	‐	63.9 (9.3)	‐
In late childhood	‐	62.8 (10.4)	63.0 (10.4)	62.8 (10.4)
Age at follow‐up (years)				
In infancy	0.5 (0.1)	‐	0.5 (0.1)	‐
In late childhood	‐	9.4 (0.8)	9.4 (0.8)	9.4 (0.8)

Abbreviations: BRLM, Bayesian Relevant Lifecourse Exposure Model; CES‐D, Center for Epidemiologic Studies Depression Scale; PSS, Perceived Stress Scale; SD, standard deviation; STAI, State‐Trait Anxiety Inventory State Version.

### Maternal psychological distress during pregnancy and child temperament traits in infancy and late childhood

A higher maternal psychological distress PCA score during pregnancy was significantly associated with higher negative affectivity and lower effortful control in infancy and in late childhood and with lower extraversion in infancy (Table 2). These associations were independent of all maternal, sociodemographic and perinatal covariates in models 1–2. Maternal concurrent distress PCA score was also associated with higher negative affectivity and lower effortful control at both follow‐ups and with lower extraversion in infancy (Table S5).

**TABLE 2:** Maternal psychological distress during pregnancy and offspring temperament traits in infancy and late childhood. The results of linear regression analyses of maternal psychological distress PCA score of depressive, anxiety and perceived stress symptoms during pregnancy and child temperament traits in infancy and late childhood. Regression coefficients (*B*), 95% CI and *p*‐values. Independent variables are expressed in standard deviation and dependent variables in percentage units.

Maternal psychological distress PCA score during pregnancy	Child temperament trait					
	Negative affectivity		Extraversion		Effortful control	
	*B* (95% CI)^a^	*p*	*B* (95% CI)^a^	*p*	*B* (95% CI)^a^	*p*
Temperament in infancy^b^						
Model 1 (*n* = 2538)^c^	2.45 (2.05; 2.85)	<.001	−0.53 (−0.89; −0.17)	.004	−1.14 (−1.50; −0.77)	<.001
Model 2 (*n* = 2527)^d^	2.40 (1.99; 2.80)	<.001	−0.65 (−1.02; −0.28)	<.001	−1.25 (−1.62; −0.88)	<.001
Model 3 (*n* = 2524)^e^	0.48 (−0.04; 1.00)	.07	−0.05 (−0.53; 0.44)	.85	0.14 (−0.33; 0.62)	.55
Temperament in late childhood^b^						
Model 1 (*n* = 2004)^c^	3.54 (3.00; 4.08)	<.001	0.01 (−0.51; 0.53)	.97	−2.59 (−3.02; −2.16)	<.001
Model 2 (*n* = 1999)^d^	3.55 (3.00; 4.09)	<.001	−0.00 (−0.54; 0.43)	.99	−2.49 (−2.93; −2.06)	<.001
Model 3 (*n* = 1971)^e^	1.97 (1.36; 2.58)	<.001	−0.24 (−0.85; 0.37)	.44	−1.52 (−2.02; −1.03)	<.001

*Note*: PCA score = Principal component analyses score from principal component analyses of maternal depressive, anxiety and perceived stress symptom levels during pregnancy. Expressed in standard deviation units. Child temperament trait scores are expressed in percentage units. A higher score indicates a score closer to the maximum.

Abbreviations: CI, confidence intervals; PCA score, principal component analyses score.

^a^ Unstandardized regression coefficients (B) and their 95% CI.

^b^ The results of linear regression analyses on maternal psychological distress during pregnancy PCA score and child temperament traits in infancy and in late childhood, respectively.

^c^ Model 1 is adjusted for child age and sex.

^d^ Model 2 is adjusted for maternal education, age at delivery, parity (primiparous vs. other), substance use (alcohol or smoking) in early pregnancy, cardiometabolic pregnancy disorders (diabetes disorders, overweight/obesity, and hypertensive pregnancy disorders) and maternal lifetime history of mental and behavioral disorders, and child sex, age, preterm versus term birth and birth weight.

^e^ Model 3 is adjusted for maternal concurrent psychological distress PCA score, maternal education, age at delivery, parity (primiparous vs. other), substance use (alcohol or smoking) in early pregnancy, cardiometabolic pregnancy disorders and lifetime history of mental and behavioral disorders, and child sex, age, preterm versus term birth and birth weight.

After adjustment for maternal concurrent distress, the associations of maternal distress during pregnancy with infant temperament traits were no longer significant (Table 2). In contrast, in late childhood, maternal distress PCA score during pregnancy was associated with higher negative affectivity and lower effortful control in children independently of maternal concurrent distress (Table 2).

The associations of maternal concurrent distress with child temperament were independent of maternal distress during pregnancy (Table S5).

### Maternal psychological distress during pregnancy and change in child temperament from infancy to late childhood

**FIGURE 1:**
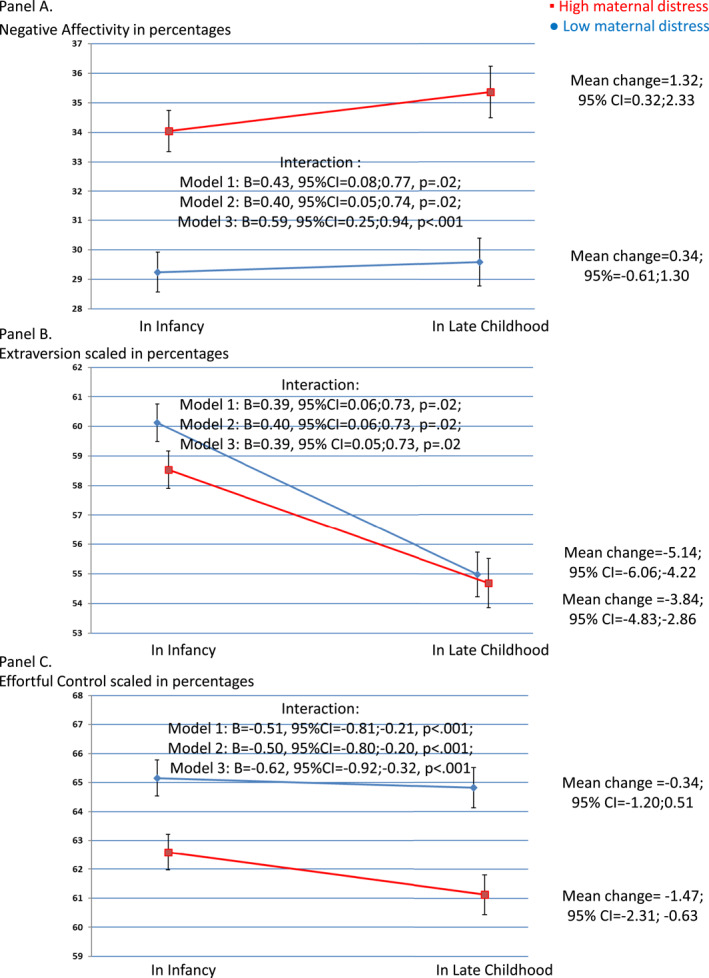
Maternal psychological distress during pregnancy and change in child temperament traits from infancy to late childhood. The figure shows the mean scores of child negative affectivity (A), extraversion (B) and effortful control (C) in infancy and in late childhood in groups divided by the median value of maternal psychological distress during pregnancy PCA score. The scores of children of mothers with high psychological distress during pregnancy (at or above the median score) are depicted with red lines and the scores of children of mothers with low psychological distress PCA score during pregnancy (below the median score) are illustrated with blue lines. The mean differences and 95% CIs show the change in temperament trait scores from infancy to late childhood in each group. Child temperament trait scores are expressed in percentages. A higher score indicates a score that is closer to the maximum. The unstandardized coefficient estimates (B), their 95% CIs and *p*‐values for interaction show the results of the linear mixed model analyses of the interaction between maternal psychological distress during pregnancy PCA score and child age on child temperament traits. Model 1 is adjusted for child sex and includes the main effects of child age and maternal psychological distress PCA score during pregnancy, along with their interaction effect. Model 2 is adjusted also for maternal education, substance use during pregnancy, parity, age, cardiometabolic pregnancy disorders and mental and behavioral disorders and child gestational age and birth weight. Model 3 is adjusted further for maternal concurrent psychological distress at the time of child temperament assessment. We used compound symmetry covariance type for our repeated measure variable child age at follow‐up. Child age is expressed in standard deviation units (standardized in the long format data). CIs, confidence intervals; PCA, principal component analysis.

Mixed model interaction analyses illustrated in Figure 1 show that maternal psychological distress PCA score during pregnancy was associated with change in child temperament from infancy to late childhood. These associations of maternal distress during pregnancy with change in child temperament traits from infancy to late childhood were independent of maternal, perinatal and sociodemographic covariates and of maternal concurrent distress (Figure 1). In children of mothers who scored at or above the median on the distress PCA score during pregnancy, negative affectivity (Figure 1A) scores increased and effortful control (Figure 1C) scores decreased significantly from infancy to late childhood. In contrast, in children of mothers who scored below the median in the distress PCA score during pregnancy there were no significant changes in these traits (Figure 1A,C). Extraversion scores decreased significantly in both groups, but less in children of mothers who scored at or above the median in the distress PCA score during pregnancy (Figure 1B).

### Sensitive exposure periods of maternal psychological distress on child temperament traits in late childhood

Table S6 shows that among the participants with distress data at all developmental periods, maternal psychological distress PCA scores during pregnancy, infancy and late childhood were associated with higher negative affectivity and lower effortful control in late childhood. In contrast, the maternal distress PCA scores at any developmental period did not show significant associations with child extraversion in late childhood (Table S6).

Consequently, BRLM analyses showed a lifetime‐to‐date effect of maternal psychological distress on higher negative affectivity (Δ = 5.01, 95% CrI = 4.38; 5.63) and lower effortful control (Δ = −3.45, 95% CrI = −3.97; −2.94) in children in late childhood. There was no lifetime‐to‐date effect on late childhood extraversion (Δ = 0.25, 95% CrI = −0.41; 0.92).

**FIGURE 2:**
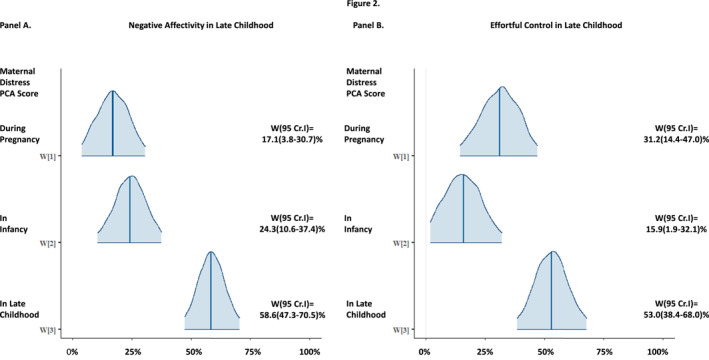
Sensitive periods and relative weights of different exposure periods for the lifetime effects of maternal psychological distress on child negative affectivity (A) and effortful control (B) in late childhood. The figure depicts the relative weights (W) and their 95% Credible Intervals (CrI) from BRLM, which indicate the relative contributions of maternal psychological distress during pregnancy, in infancy and in late childhood to the lifetime effects of maternal psychological distress on child negative affectivity (A) and effortful control (B) in late childhood. The BRLM analyses are adjusted for child age and sex. The weights are expressed in percentages, with a possible minimum of 0% indicating no influence and a maximum of 100% indicating a possible critical period. A higher weight indicates a larger contribution to the lifetime effect. BRLM, Bayesian Relevant Lifecourse Exposure Model.

Of the temperament traits with lifetime‐to‐date effects of maternal psychological distress, Figure 2 shows that, for both negative affectivity (A) and effortful control (B), maternal distress in late childhood had the highest weights/contribution (Figure 2). This suggested that late childhood was the sensitive period for the effects of maternal distress on both traits. Indeed, the models suggested that late childhood was the sensitive period and had a 0.15 Euclidean distance and an over 99.9% posterior probability for negative affectivity and a 0.23 Euclidean distance and a 94.7% posterior probability for effortful control (Table S7).

### Does child sex modify the associations maternal psychological distress with child temperament traits?

We found no interactions of maternal psychological distress during pregnancy PCA score with child sex on child temperament traits in infancy or late childhood or on change in temperament traits from infancy to late childhood (all *p*‐values ≥ .06). There were neither any interaction effects of maternal psychological distress and child sex on the lifetime‐to‐date effects on temperament traits in late childhood (all CrI:s crossing zero).

## DISCUSSION

In our large, prospective study, maternal psychological distress during pregnancy was associated with higher negative affectivity and lower effortful control in children in infancy and late childhood, with lower extraversion in infancy and with changes in negative affectivity, extraversion and effortful control from infancy to late childhood. All these associations were independent of several maternal, sociodemographic, and perinatal covariates. The associations with child temperament traits in late childhood and with temperament change from infancy to late childhood were also independent of maternal concurrent psychological distress. Nevertheless, late childhood was a sensitive period for the lifetime‐to‐date effects maternal psychological distress had on late childhood temperament, while distress during pregnancy and child's infancy had smaller influences. Child sex did not modify the associations of maternal distress with child temperament.

Our findings in infants correspond with previous findings of associations between maternal psychological distress during pregnancy and higher negative affectivity (Rogers et al., 2020; Spry, Aarsman, et al., 2020) and lower effortful control (Rogers et al., 2020). We also found associations with lower infant extraversion, while previous findings were inconclusive (Erickson et al., 2017). However, these associations of maternal distress during pregnancy with infant temperament were not independent of maternal concurrent distress. Also previous findings on the independence of these associations were inconclusive (Korja et al., 2017). This may reflect a more relevant role for maternal concurrent than prenatal distress for infant temperament. It may also reflect the high continuity of maternal distress from pregnancy to child's infancy and that maternal distress during pregnancy exerts effects on infant temperament traits only indirectly via maternal concurrent distress.

In late childhood, our findings of associations of maternal psychological distress during pregnancy with higher negative affectivity and lower effortful control in children correspond with most of the previous studies on child temperament after 3 years of age (Glynn et al., 2018; Mahrer et al., 2020; Rinne et al., 2022; Sutin et al., 2022; Swales et al., 2023). The previous prospective studies all had small sample sizes (Glynn et al., 2018; Mahrer et al., 2020; Rinne et al., 2022; Swales et al., 2023). In the one larger study, maternal distress was assessed retrospectively (Sutin et al., 2022). Ours is thus the largest prospective study on maternal distress during pregnancy and late childhood temperament. The associations of maternal distress during pregnancy with late childhood temperament were independent of maternal concurrent distress. This may reflect the larger effect sizes maternal distress during pregnancy had on child temperament traits in late childhood than in infancy, the more direct effects of maternal distress during pregnancy on child temperament traits at this later developmental stage and/or the lower continuity of distress from pregnancy to late childhood than to infancy.

Our novel findings on temperament change showed that higher maternal psychological distress during pregnancy was associated with an increase in negative affectivity, a decrease in effortful control and a smaller decrease in extraversion from infancy to late childhood, independently of maternal concurrent distress. The previous retrospective study showed no associations between maternal distress during pregnancy and change in child temperament from 4–7 to 8–15 years of age (Sutin et al., 2022). Our study is the first on maternal distress during pregnancy and temperament change including infancy, the developmental period when temperament changes the most (Briley & Tucker‐Drob, 2014; Putnam et al., 2008). That maternal distress during pregnancy was also independently associated with change in child temperament may possibly indicate a more causal effect underlying these associations (McCrae & Sutin, 2018). However in epidemiological studies, causality deductions are preliminary at best (Ramspek et al., 2021). The patterns of temperament change that were observed between infancy and late childhood in children of mothers with high psychological distress during pregnancy made the differences in negative affectivity and effortful control, which were observed already in infancy, stronger and more manifest in late childhood. In contrast, they became nonexistent for extraversion. The two traits with the highest relevance for developmental psychopathology thus showed stronger associations with maternal distress during pregnancy as the traits became more developed (Kostyrka‐Allchorne et al., 2020). It is possible that evocative, active and passive correlations between child's temperament traits and the environments the child develops in underlie the associations with temperament change and make the associations between maternal psychological distress and child temperament stronger as the child develops (Perlstein & Waller, 2022). However, the effect sizes of maternal distress during pregnancy on temperament change were relatively small, as were the average within‐individual changes in temperament traits from infancy to late childhood.

We were the first to examine sensitive exposure periods for the associations of maternal psychological distress with late childhood temperament. Maternal distress had lifetime‐to‐date effects on higher negative affectivity and lower effortful control in children. Late childhood was a sensitive exposure period with over 50% relative contributions for the lifetime‐to‐date effects on both traits, but maternal distress during pregnancy and infancy also had smaller contributions. The relevant role of distress in all periods was highlighted by the accumulative effect model being the second best fitting model for both traits.

Overall, our findings suggest a consistent temperamental phenotype of high negative affectivity and low effortful control, indicating increased vulnerability to stress, in children exposed to maternal psychological distress during and after pregnancy (Pluess & Belsky, 2011). The life cycle model of stress suggests that maternal distress leads to increased vulnerability to stress in children (Lupien et al., 2009). Temperamentally, this vulnerability is evident in high negative affectivity and low effortful control: a child with these traits persistently experiences high sadness, anger and fear, has difficulties in regulating these emotions, and is at an increased risk of psychiatric problems (Kostyrka‐Allchorne et al., 2020; Pluess & Belsky, 2011; Rothbart, 2007). Evidence also suggests that maternal distress may be associated with child temperament traits via altered neurobiological stress sensitivity, as indicated by possibly epigenetic changes in hypothalamus‐pituitary‐adrenal‐cortex‐axis, inflammatory and autonomic nervous system functioning (Davis & Narayan, 2020; Gartstein & Skinner, 2018; Gustafsson et al., 2018; Van den Bergh et al., 2020). The associations of maternal distress with child temperament may also reflect the shared genetic risk factors between maternal depressive, anxiety and perceived stress symptoms and temperament traits in children (Ahmadzadeh et al., 2021; Mikolajewski et al., 2013). Indeed, genetically informed studies suggest that shared genetic factors at least partially explain the associations of maternal distress with child psychological development (Ahmadzadeh et al., 2021; Chen et al., 2023; Hannigan et al., 2018). Future studies specifically on child temperament with genetically informed designs are needed.

The mechanisms underlying the associations of maternal psychological distress during pregnancy, child's infancy and late childhood with child temperament traits may be partially similar to and partially different from each other. While shared genetic risks probably contribute to the associations in all the exposure periods, teratogenic effects of maternal distress during pregnancy on fetal brain development offer another plausible mechanism for the associations of maternal distress during pregnancy with child temperament (Kling et al., 2023; Van den Bergh et al., 2020). Further, the associations with maternal distress in offspring's infancy and late childhood may also reflect transactional associations of maternal distress, child temperament, parenting behaviors, and mother‐child attachment, since meta‐analyses show that the security of mother‐child attachment and parenting behaviors are associated with maternal distress and with child temperament (Barnes & Theule, 2019; Goodman et al., 2020; Pallini et al., 2018; Paulussen‐Hoogeboom et al., 2007). Furthermore, research shows that these associations may be transactional, with both mother‐to‐child effects and evocative child‐to‐mother influences (Chad‐Friedman et al., 2023; Hentges et al., 2021; Paulussen‐Hoogeboom et al., 2007). The underlying neurobiological mechanisms for the effects of maternal distress in all exposure periods may include altered neurobiological stress vulnerability, genetic vulnerabilities and epigenetic changes in gene expression, since such changes have been found in association with maternal distress during pregnancy, parenting behaviors and the security of parent‐child attachment (Alen et al., 2022; Brien et al., 2023; Measelle et al., 2017). The findings of late childhood being a sensitive exposure period for the effects of maternal distress on child temperament may reflect transactional mother‐to‐child and child‐to‐mother associations between maternal distress and child temperament (Perlstein & Waller, 2022). On the other hand, these sensitive exposure period ‐findings may also reflect recency effects, since evidence shows that maternal most recent distress has the strongest influence on parenting and child development (Goodman et al., 2020). Further studies should elucidate whether late childhood is a sensitive exposure period for the effects of maternal distress on child temperament also later in child's development (Gabard‐Durnam & Mclaughlin, 2019).

It is also possible that concurrent psychological distress would be influencing mother's rating of her child's temperament the most (Lo et al., 2015). Indeed the limitations of our study include lack of data from fathers and using maternal reports of both maternal distress and child temperament, inducing bias related to shared method variance and to maternal mood possibly influencing the ratings of her child (Lo et al., 2015). Such a bias may overestimate associations between maternal distress at any developmental period and child temperament. However, psychometric evidence suggests that rater bias effects on the associations between maternal distress and child temperament are very small (Olino et al., 2020). Furthermore, parent‐reports of temperament traits according to the psychobiological framework have good ecological and predictive validity (Gartstein & Rothbart, 2003; Kostyrka‐Allchorne et al., 2020). Nevertheless, further studies on maternal distress and child temperament would benefit from combining biparental temperament ratings with teacher and laboratory assessments of child temperament (Lo et al., 2015; Putnam & Stifter, 2008). Other factors limiting the generalizability of our findings include attrition selective to maternal health and our sample comprising participants within a quite prosperous Nordic country. Since the participating women had lower anxiety and perceived stress levels during pregnancy and fewer of them had lifetime‐to‐date mental disorders than the non‐participating women, this selective attrition may have biased our findings. While selective attrition can either increase or decrease the ability to detect associations between exposures and outcomes, the associations we found were highly statistically significant and thus unlikely to solely reflect attrition bias. However, our findings best generalize to a population that does not differ in the abovementioned characteristics from ours. Furthermore, our findings in late childhood need replication in other societies, although maternal distress is associated with infant temperament also in other cultures (Chong et al., 2016; Spry, Aarsman, et al., 2020). The age ranges of the children for the two temperament follow‐ups were relatively wide: over 8 months in infancy and 4 years in late childhood. However, all children were of the appropriate age for the temperament scales used, and 80% of children were within a 1.5 months age range in infancy and a 2 years age range in late childhood. Importantly, we accounted for this slight between‐person variation by adjusting for child age in all our statistical analyses, thus taking into account any bias related to age variation. Moreover, in spite of these between‐person variations in child age, the within‐person variation in maternal distress and child temperament ratings in infancy and late childhood was minimal, since the mothers reported their distress levels concurrently to child temperament ratings. On the other hand, the strengths of our study include its prospective design, the large sample size and the repeated, validated assessments of maternal psychological distress and child temperament.

## CONCLUSION

Maternal psychological distress during pregnancy is associated with higher negative affectivity and lower effortful control in children in infancy and late childhood and with change in child temperament from infancy to late childhood. The associations with child temperament traits in late childhood and change in child temperament from infancy to late childhood are independent of maternal concurrent psychological distress. Maternal psychological distress has lifetime‐to‐date effects on late childhood temperament, to which maternal distress in late childhood contributes the most, but distress during pregnancy and infancy also affect.

## AUTHOR CONTRIBUTIONS

**Marius Lahti‐Pulkkinen**: Conceptualization; data curation; formal analysis; funding acquisition; investigation; methodology; project administration; software; validation; visualization; writing – original draft; writing – review & editing. **Anna Lähdepuro**: Formal analysis; funding acquisition; investigation; visualization; writing – review & editing. **Jari Lahti**: Funding acquisition; investigation; methodology; project administration; writing – review & editing. **Polina Girchenko**: Conceptualization; formal analysis; investigation; methodology; writing – review & editing; **Riikka Pyhälä**: Conceptualization; investigation; methodology; writing – review & editing. **Rebecca M. Reynolds**: Conceptualization; investigation; methodology; writing – review & editing. **Pia M. Villa**: Conceptualization; investigation; project administration; resources; writing – review & editing. **Hannele Laivuori**: Conceptualization; funding acquisition; investigation; methodology; resources; writing – review & editing. **Eero Kajantie**: Conceptualization; funding acquisition; investigation; methodology; resources; writing – review & editing. **Kati Heinonen**: Conceptualization; funding acquisition; methodology; project administration; writing – review & editing. **Katri Räikkönen**: Conceptualization; funding acquisition; investigation; methodology; project administration; resources; validation; visualization; writing – review & editing.

## CONFLICT OF INTEREST STATEMENT

The authors have declared that they have no competing or potential conflicts of interest.

## ETHICAL CONSIDERATIONS

The data are not publicly available due to privacy or ethical restrictions. Since our study data includes sensitive patient data, we cannot make it open access. Researchers interested in data access may contact the PREDO study board. Data requests will be subject to further reviews by relevant register authorities.

## Supporting information

Supporting Information S1

## Data Availability

The data that support the findings of this study are available on request from the corresponding author.
