# Supplementary material for: Maternal psychological distress and temperament traits in children from infancy to late childhood
Source: JCPP Adv. 2024 May 6;4(3):e12242. doi: 10.1002/jcv2.12242 (PMC11472812; doi:10.1002/jcv2.12242)
Supplement: Supplementary file 1 — Supporting Information S1 [file JCV2-4-e12242-s001.pdf]

**Table S1. Attrition Analyses**. Differences between cohort members excluded from the current study on key sample characteristics compared to the different subsamples of the current study sample. Means and standard deviations for continuous covariates and number and percentage of participants for categorical covariates. P-values from t-tests and $\chi^2$-tests, comparing the participants of a certain subsample to the mothers or children excluded from the current study in the distributions of the characteristic in question.

| | **Excluded from the Current Study** | **Has Temperament Data in Infancy + Distress Data during Pregnancy** | | **Has Temperament Data in Late Childhood + Distress Data during Pregnancy** | | **Has Temperament Data in Infancy and Late Childhood+ Distress Data During Pregnancy** | | **Has Temperament Data in Late Childhood + Data on Distress during Pregnancy, in Infancy and in Late Childhood** | |
|---|---|---|---|---|---|---|---|---|---|
| N | 1925 | 2538 | | 2004 | | 1693 | | 1926 | |
| **Maternal Characteristics** | Mean(SD)/ N(%) | Mean(SD)/ N(%) | p | Mean(SD)/ N(%) | p | Mean(SD)/ N(%) | p | Mean(SD)/ N(%) | p |
| Psychological Distress During Pregnancy | | | | | | | | | |
| CES-D Depressive Symptoms | 11.6(6.4) | 11.4(6.4) | .57 | 11.2(6.3) | .18 | 11.2(6.2) | .16 | 11.2(6.2) | .16 |
| STAI Anxiety Symptoms | 33.5(7.9) | 33.0(7.7) | .22 | 32.7(7.4) | .04 | 32.7(7.5) | .05 | 32.7(7.4) | .04 |
| PSS Perceived Stress Symptoms | 5.6(2.6) | 5.2(2.5) | .002 | 5.1(2.4) | <.001 | 5.1(2.4) | <.001 | 5.1(2.4) | <.001 |
| Data Missing | 1375-1377 | | | | 0 | | | | |
| Age at Delivery (years) | 31.0(5.2) | 31.9(4.7) | <.001 | 32.0(4.5) | <.001 | 32.0(4.5) | <.001 | 32.0(4.5) | <.001 |
| Data Missing | 10 | | | | 0 | | | | |
| Education Level | | | | | | | | | |
| Primary or Secondary | 706(44.5%) | 774(30.5%) | <.001 | 506(25.2%) | <.001 | 421(24.9%) | <.001 | 489(25.4%) | <.001 |
| Lover Tertiary | 881(55.5%) | 1764(69.5%) | | 1498(74.8%) | | 1272(75.1%) | | 1437(74.6%) | |
| Data Missing | 338 | | | | 0 | | | | |
| Parity | | | | | | | | | |
| Primiparous | 683(35.5%) | 1070(42.2%) | <.001 | 1187(40.8%) | <.001 | 715(42.2%) | <.001 | 791(41.1%) | <.001 |
| Multiparous | 1242(64.5%) | 1468(57.8%) | | 1187(59.2%) | | 978(57.8%) | | 1135(58.9%) | |
| Cardiometabolic Pregnancy Disorders | | | | | | | | | |
| Diabetes, Hypertension, or Overweight/Obesity in Current Pregnancy | 867(45.3%) | 1078(42.5%) | .01 | 814(40.6%) | .002 | 687(40.6%) | <.001 | 783(40.7%) | .002 |
| Diabetes or Hypertension in Previous Pregnancy | 93(4.9%) | 96(3.7%) | | 80(4.0%) | | 61(3.6%) | | 74(3.8%) | |
| No Disorder | 956(49.9%) | 1366(53.9%) | | 1110(55.4%) | | 945(55.8%) | | 1069(55.5%) | |
| Data Missing | 9 | | | | 0 | | | | |
| Lifetime Mental Disorders by 31/12/2018 | | | | | | | | | |
| Yes | 429(22.5%) | 407(16.0%) | <.001 | 283(14.1%) | <.001 | 249(14.7%) | <.001 | 272(14.1%) | <.001 |
| No | 1478(77.5%) | 2129(84.0%) | | 1721(85.9%) | | 1444(85.3%) | | 1654(85.9%) | |
| Data Missing | 18 | 2 | | | | 0 | | | |

| | | | | | | | | | |
|---|---|---|---|---|---|---|---|---|---|
| **Child Characteristics** | | | | | | | | | |
| Gestational Age | | | | | | | | | |
| Preterm Birth | 89(4.6%) | 92(3.6%) | .09 | 75(3.7%) | .16 | 63(3.7%) | .17 | 71(3.7%) | .14 |
| Other (≥37) weeks) | 1832(95.4%) | 2446(96.4%) | | 1929(96.3%) | | 1630(96.3%) | | 1855(96.3%) | |
| Data Missing | 4 | | | | 0 | | | | |
| Birth Weight (kilograms) | 3.5(0.5) | 3.5(0.5) | .23 | 3.5(0.5) | .31 | 3.5(0.5) | .35 | 3.5(0.5) | .35 |
| Data Missing | 22 | 9 | | 5 | | 3 | | 3 | |
| Sex | | | | | | | | | |
| Girl | 892(46.8%) | 1241(48.9%) | .16 | 987(49.3%) | .12 | 847(50.0%) | .05 | 956(49.6%) | .07 |
| Boy | 1016(53.2%) | 1297(51.1%) | | 1017(50.7%) | | 846(50.0%) | | 970(50.4%) | |
| Data Missing | 17 | | | | 0 | | | | |

SD=Standard Deviation; CES-D: Center for Epidemiologic Studies Depression Scale; STAI: State-Trait Anxiety Inventory State Version; PSS: Perceived Stress Scale.

**Table S2. Principal Component Analyses of Maternal Psychological Distress, Assessed with Scales on Depressive, Anxiety, and Perceived Stress Symptoms.** All analyses yielded one-component solutions described below. PCA analyses for distress during pregnancy were conducted both among all participants with child temperament data in infancy and in late childhood, and among participants with distress data during pregnancy, during infancy and during late childhood and with child temperament data in late childhood.

| Maternal Psychological Distress | Component Loading (r) | Squared Component Loading ($r^2$) | Eigenvalue of the Component | Amount of Variance Explained |
|---|---|---|---|---|
| **Among participants with temperament data either during infancy or in late childhood** | | | | |
| **During Pregnancy** | | | 2.653 | 88.4% |
| Depressive Symptoms | 0.945 | 0.893 | | |
| Anxiety Symptoms | 0.940 | 0.883 | | |
| Perceived Stress Symptoms | 0.937 | 0.877 | | |
| **During Child's Infancy** | | | 2.419 | 80.6% |
| Depressive Symptoms | 0.913 | 0.834 | | |
| Anxiety Symptoms | 0.900 | 0.811 | | |
| Perceived Stress Symptoms | 0.880 | 0.775 | | |
| **During Late Childhood** | | | 2.347 | 78.2% |
| Depressive Symptoms | 0.900 | 0.810 | | |
| Anxiety Symptoms | 0.884 | 0.782 | | |
| Perceived Stress Symptoms | 0.869 | 0.755 | | |
| **Among participants eligible for the sensitive period analyses** | | | | |
| **Maternal Psychological Distress** | Component Loading (r) | Squared Component Loading ($r^2$) | Eigenvalue of the Component | Amount of Variance Explained |
| **During Pregnancy** | | | 2.649 | 88.3% |
| Depressive Symptoms | 0.945 | 0.892 | | |
| Anxiety Symptoms | 0.938 | 0.879 | | |
| Perceived Stress Symptoms | 0.937 | 0.878 | | |
| **During Child's Infancy** | | | 2.421 | 80.7% |
| Depressive Symptoms | 0.915 | 0.838 | | |
| Anxiety Symptoms | 0.900 | 0.811 | | |
| Perceived Stress Symptoms | 0.879 | 0.772 | | |
| **During Late Childhood** | | | 2.351 | 78.4% |
| Depressive Symptoms | 0.901 | 0.811 | | |
| Anxiety Symptoms | 0.886 | 0.784 | | |
| Perceived Stress Symptoms | 0.869 | 0.755 | | |

Depressive symptoms were assessed with the Center for Epidemiologic Studies Depression Scale, anxiety symptoms with the State Trait Anxiety Inventory State Version and perceived stress symptoms with the Perceived Stress Scale. Before running the principal component analyses, all scales were square root transformed and thereafter standardized.

**Table S3. The Correlations Between Temperament Traits with Each Other and Across Time.**

| Child Temperament Traits | In Infancy | | | | | | In Late Childhood | | | | | |
|---|---|---|---|---|---|---|---|---|---|---|---|---|
| | Negative Affectivity | | Extraversion | | Effortful Control | | Negative Affectivity | | Extraversion | | Effortful Control | |
| | r | p | r | p | r | p | r | p | r | p | r | p |
| **In Infancy** | | | n=2538 | | | | | | n=1693 | | | |
| Negative Affectivity | | | .03 | .15 | -.30 | <.001 | .23 | <.001 | .01 | .83 | -.12 | <.001 |
| Extraversion | | | | | .42 | <.001 | -.06 | .02 | .13 | <.001 | .13 | <.001 |
| Effortful Control | | | | | | | -.11 | <.001 | .02 | .37 | .19 | <.001 |
| **In Late Childhood** | | | | | | | | | n=2004 | | | |
| Negative Affectivity | | | | | | | | | -.01 | .81 | -.40 | <.001 |
| Extraversion | | | | | | | | | | | -.21 | <.001 |
| Effortful Control | | | | | | | | | | | | |

**Table S4. The Associations Between Covariates and Temperament Traits in Children.** Regression coefficients and 95% Confidence Intervals from age-specific linear regressions in predicting the temperament trait in question. Statistically significant associations are shown in boldface. Temperament traits are expressed in percentage units and continuous covariates are expressed in standard deviation units.

| Child Temperament Trait | Negative Affectivity | | Extraversion | | Effortful Control | |
|---|---|---|---|---|---|---|
| | In Infancy | In Late Childhood | In Infancy | In Late Childhood | In Infancy | In Late Childhood |
| | B(95%CI) | B(95%CI) | B(95%CI) | B(95%CI) | B(95%CI) | B(95%CI) |
| **Maternal Characteristic** | | | | | | |
| Age | **-0.79(-1.22;-0.35)** | -0.43(-1.02;0.17) | -0.29(-0.67;0.10) | -0.49(-1.05;0.07) | **0.84(0.46;1.22)** | **0.53(0.04;1.02)** |
| Parity: Primi- vs. Multiparous (ref.) | **-0.84(-1.68;-0.00)** | **2.98(1.86;4.09)** | 0.07(-0.67;0.82) | -0.55(-1.61:0.51) | -0.50(-1.25;0.24) | -0.24(-1.17;0.69) |
| Education | | | | | | |
| Primary or Secondary | -0.65(-1.56:0.27) | **1.37(0.07;2.67)** | **2.41(1.60;3.24)** | **1.41(0.20;.2.62)** | **1.81(1.00;2.62)** | **-2.50(-3.58;-1.42)** |
| Tertiary | Ref. | | | | | |
| Substance use during Early Pregnancy: Yes vs. No (ref.) | -0.38(-1.42;0.65) | -0.03(-1.41;1.35) | -0.83(-1.74;0.09) | 0.52(-0.78;1.82) | -0.65(-1.57;0.27) | -0.93;-2.07;0.21) |
| Lifetime Mental Disorders: yes/ no | **2.72(1.24;4.20)** | **4.08(2.49;5.68)** | 0.12(-1.19;1.43) | -0.70(-2.21;0.81) | -0.31(-1.62;1.01) | **-2.75(-4.08;-1.44)** |
| Cardiometabolic Pregnancy Disorder | | | | | | |
| Diabetes, Hypertensive Disorder or Overweight/Obesity in Index Pregnancy | 0.29(-0.56;1.14) | -0.14(-1.29;1.00) | **1.43(0.68;2.18)** | 0.90(-0.17:1.98) | **0.91(0.16;1.67)** | -0.37(-1.31;0.58) |
| Diabetes or Hypertension Before Current Pregnancy Only | 0.34(-1.87;2.55) | -2.63(-5.45;0.19) | -0.07(-2.02;1.88) | -0.36(-3.05;2.33) | 1.96(-0.02;3.94) | 1.34(-1.00;3.67) |
| No Pregnancy Disorder | Ref. | | | | | |
| **Child Characteristic** | | | | | | |
| Age | **1.18(0.77;1.59)** | **-0.93(-1.48;-0.38)** | **0.93(0.56;1.29)** | **-0.87(-1.39;-0.35)** | **-0.86(-1.23;-0.49)** | **-0.37(-0.83;0.08)** |
| Gender: Girl vs. Boy (ref) | -0.08(-0.91;0.75) | 0.83(-0.27;1.94) | **-1.15(-1.88;-0.42)** | **-4.17(-5.19;-3.14)** | 0.52(-0.21;1.26) | **6.29(5.42;7.16)** |
| Preterm Birth: Yes vs. no | 0.37(-1.84;2.58) | 2.06(-0.85;4.97) | **-3.01(-4.96:-1.05)** | 0.78(-1.97;3.52) | 0.24(-1.73;2.20) | -2.07(-4.48;0.33) |
| Birth weight | 0.40(-0.02;0.83) | -0.25(-0.82;0.32) | **0.73(0.36;1.11)** | -0.02(-0.55;0.52) | 0.02(-0.36;0.40) | -0.11(-0.57;0.36) |

**Table S5. Maternal Concurrent Psychological Distress and Offspring Temperament Traits in Infancy and Late Childhood.** The results of linear regression analyses of maternal concurrent psychological distress principal component analysis (PCA) score of depressive, anxiety and perceived stress symptoms and child temperament traits in infancy and late childhood. Regression coefficients (B), 95% confidence intervals (CI) and p-values. Independent variables are expressed in standard deviation and dependent variables in percentage units.

| | Child Temperament Trait | | | | | |
|---|---|---|---|---|---|---|
| | Negative Affectivity | | Extraversion | | Effortful Control | |
| **Maternal Concurrent Psychological Distress PCA score** | **B(95% CI)[a]** | **p** | **B(95% CI)[a]** | **p** | **B(95% CI)[a]** | **p** |
| **Temperament in Infancy[b]** | | | | | | |
| Model 1 (n=2535)[c] | 3.32(2.93;3.71) | <.001 | -0.79(-1.16;-0.43) | <.001 | -1.92(-2.28;-1.56) | <.001 |
| Model 2 (n=2524)[d] | 3.27(2.87;3.66) | <.001 | -0.94(-1.31;-0.58) | <.001 | -2.03(-2.39;-1.66) | <.001 |
| Model 3 (n=2524)[e] | 2.96(2.44;3.48) | <.001 | -0.91(-1.39;-0.43) | <.001 | -2.12(-2.60;-1.64) | <.001 |
| **Temperament in Late Childhood[b]** | | | | | | |
| Model 1 (n=1976)[c] | 4.28(3.75;4.80) | <.001 | 0.32(-0.19;0.83) | .22 | -2.77(-3.19;-2.35) | <.001 |
| Model 2 (n=1971)[d] | 4.10(3.57;4.63) | <.001 | 0.34(-0.18;0.87) | .20 | -2.66(-3.09;-2.23) | <.001 |
| Model 3 (n=1971)[e] | 3.15(2.55;3.75) | <.001 | 0.46(-0.14;1.06) | .14 | -1.92(-2.41;-1.43) | <.001 |

PCA score=Principal Component Analyses score from principal component analyses of maternal depressive, anxiety and perceived stress symptom levels concurrently to rating the child temperament. Expressed in standard deviation units. Child temperament trait scores are expressed in percentage units. A higher score indicates a score closer to the maximum.

[a] Unstandardized regression coefficients (B) and their 95% confidence intervals (CI).

[b] The results of linear regression analyses on maternal psychological distress during pregnancy PCA score and child temperament traits in infancy and in late childhood, respectively.

[c]Model 1 is adjusted for child age and sex.

[d]Model 2 is adjusted for maternal education, age at delivery, parity (primiparous vs. other), substance use (alcohol or smoking) in early pregnancy, cardiometabolic pregnancy disorders (diabetes disorders, overweight/obesity, and hypertensive pregnancy disorders) and maternal lifetime history of mental and behavioral disorders, and child sex, age, preterm vs. term birth and birth weight.

[e] Model 3 is adjusted for maternal psychological distress during pregnancy PCA score, education level, age at delivery, parity, substance use in early pregnancy, cardiometabolic pregnancy disorders and lifetime history of mental and behavioral disorders, and child sex, age, preterm vs. term birth and birth weight.

**Table S6. Maternal Psychological Distress during Pregnancy, in Child's Infancy and in Late Childhood and Offspring Temperament Traits in Late Childhood among Participants with Maternal Psychological Distress Data during Pregnancy, In Infancy and in Late Childhood (n=1926).** The associations of principal component scores of maternal depressive, anxiety, and perceived stress symptoms during pregnancy, in child's infancy and in late childhood with child temperament traits in late childhood. Regression Coefficients (B) and 95% Confidence Intervals (CI) from linear regression analyses. Independent variables are expressed in standard deviation units and dependent variables are expressed in percentage units. A higher percentage score indicates a score closer to the maximum.

| Child Temperament Trait | Negative Affectivity | | | | Extraversion | | | | Effortful Control | | | |
|---|---|---|---|---|---|---|---|---|---|---|---|---|
| | B | 95% CI | | p | B | 95% CI | | p | B | 95% CI | | p |
| **Maternal Psychological Distress During Pregnancy PCA Score and Child Temperament Traits In Late Childhood[a]** | | | | | | | | | | | | |
| Model 1 | 3.52 | 2.98 | 4.06 | <.001 | -0.00 | -0.52 | 0.52 | 0.99 | -2.55 | -2.98 | -2.12 | <.001 |
| Model 2 | 3.48 | 2.94 | 4.03 | <.001 | -0.06 | -0.59 | 0.47 | 0.82 | -2.43 | -2.87 | -1.99 | <.001 |
| **Maternal Psychological Distress in Infancy PCA Score and Child Temperament Traits in Late Childhood[a]** | | | | | | | | | | | | |
| Model 1 | 3.54 | 3.00 | 4.08 | <.001 | 0.13 | -0.39 | 0.63 | 0.60 | -2.33 | -2.76 | -1.89 | <.001 |
| Model 2 | 3.41 | 2.87 | 3.96 | <.001 | 0.09 | -0.44 | 0.62 | 0.74 | -2.19 | -2.63 | -1.75 | <.001 |
| **Maternal Psychological Distress in Late Childhood PCA Score and Child Temperament Traits in Late Childhood[a]** | | | | | | | | | | | | |
| Model 1 | 4.34 | 3.82 | 4.87 | <.001 | 0.37 | -0.15 | 0.89 | 0.16 | -2.85 | -3.27 | -2.42 | <.001 |
| Model 2 | 4.13 | 3.58 | 4.67 | <.001 | 0.37 | -0.17 | 0.90 | 0.18 | -2.70 | -3.14 | -2.27 | <.001 |

PCA score=Principal Component Analyses component score from principal component analyses of levels of maternal depressive, anxiety and perceived stress symptoms. Unstandardized regression coefficients (B) and their 95% confidence intervals (CI). Maternal psychological distress PCA scores are expressed in standard deviation units. The child temperament trait scores are expressed in percentage units.

[a] The results of linear regression analyses on maternal psychological distress during pregnancy, in child's infancy and in late childhood and temperament traits in children in late childhood. Model 1 is adjusted for child age and sex. Model 2 is adjusted also for maternal education, age at delivery, parity (primiparous vs. other), substance use (alcohol or smoking) in early pregnancy, cardiometabolic pregnancy disorders (diabetes disorders, overweight/obesity, and hypertensive pregnancy disorders) and maternal lifetime history of mental and behavioral disorders, and child preterm vs. term birth and birth weight.

**Table S7. The model fits of the different Bayesian Relevant Lifecourse Exposure Models of the Effects of Maternal Psychological Distress During Pregnancy, in Child's Infancy and in Late Childhood on Child Negative Affectivity and Effortful Control in Late Childhood.** Euclidean distances and their 95% credible intervals. A shorter Euclidean distance indicates Better model fit.

| **Maternal Psychological Distress and Child Negative Affectivity** | **Euclidean Distance(95% Credible Interval)** |
|---|---|
| Pregnancy as a Sensitive Period | 0.66(0.50-0.81) |
| Infancy as a Sensitive Period | 0.60(0.45-0.75) |
| Late Childhood as a Sensitive Period | 0.15(0.03-0.28) |
| Late Childhood as a Critical Period | 0.52(0.37-0.66) |
| Accumulative Effect Model | 0.33(0.19-0.47) |
| **Maternal Psychological Distress and Child Effortful Control** | **Euclidean Distance(95% Credible Interval)** |
| Pregnancy as a Sensitive Period | 0.52(0.34-0.71) |
| Infancy as a Sensitive Period | 0.65(0.46-0.81) |
| Late Childhood as a Sensitive Period | 0.23(0.06-0.41) |
| Late Childhood as a Critical Period | 0.59(0.41-0.78) |
| Accumulative Effect Model | 0.28(0.12-0.45) |
